# Cerebral Concussion Primes the Lungs for Subsequent Neutrophil-Mediated Injury

**DOI:** 10.1097/CCM.0000000000003270

**Published:** 2018-08-15

**Authors:** Duncan C. Humphries, Stephen O’Neill, Emma Scholefield, David A. Dorward, Alison C. Mackinnon, Adriano G. Rossi, Christopher Haslett, Peter J. D. Andrews, Jonathan Rhodes, Kevin Dhaliwal

**Affiliations:** 1 MRC Centre for Inflammation Research, University of Edinburgh, Edinburgh, United Kingdom.; 2 Centre for Clinical Brain Sciences, University of Edinburgh, Edinburgh, United Kingdom.; 3 Department of Anaesthesia, Critical Care and Pain Medicine, University of Edinburgh, Western General Hospital, Edinburgh, United Kingdom.

**Keywords:** acid microaspiration, acute respiratory distress syndrome, concussion, inflammation, neutrophils, traumatic brain injury

## Abstract

**Objectives:**

Mild traumatic brain injury in the form of concussion is extremely common, and the potential effects on pulmonary priming have been underestimated. The aim of this study was to characterize the pulmonary response following mild traumatic brain injury and assess the pulmonary susceptibility to lung injury after a subsequent innocuous pulmonary insult.

**Design:**

Experimental in vivo study.

**Setting:**

University research laboratory.

**Subjects:**

Male CD1 mice.

**Interventions:**

We developed a model of concussive traumatic brain injury in mice followed by pulmonary acid microaspiration. To assess the dependent role of neutrophils in mediating pulmonary injury, we specifically depleted neutrophils.

**Measurements and Main Results:**

Lateral fluid percussion to the brain resulted in neuronal damage and neutrophil infiltration as well as extensive pulmonary interstitial neutrophil accumulation but no alveolar injury. Following subsequent innocuous acid microaspiration, augmented alveolar neutrophil influx led to the development of pulmonary hemorrhage that was reduced following neutrophil depletion.

**Conclusions:**

This model shows for the first time that innocuous acid microaspiration is sufficient to induce neutrophil-mediated lung injury following mild concussion and that the extracranial effects of mild traumatic brain injury have been underestimated.

## Abstract 2

Supplemental Digital Content is available in the text.

Damage to the CNS in the form of traumatic brain injury (TBI) can lead to multiple organ dysfunction syndrome (1). The lung is the most frequent organ to be affected, manifested by the development of acute respiratory distress syndrome (ARDS) (1, 2). ARDS occurs in 20–25% of patients with isolated brain injury (3) and is a critical independent factor affecting mortality. The mechanisms for ARDS development in this population remain to be defined. Lung-protective strategies can improve the outcome of TBI patients (4); however, life-threatening pulmonary dysfunction is extremely difficult to treat (5). Furthermore, this has major implications for lung transplantation, with less than one third of nonsurviving severe TBI patients considered suitable for lung donation, of which more than 80% fail to donate (6).

A “double hit” model has been hypothesized to explain the increased pulmonary susceptibility to injury after severe TBI (7) involving a sympathetic catecholamine storm that induces type II pneumocyte damage of the pulmonary endothelium (8). This, coupled with a systemic inflammatory response induced by increased intracranial proinflammatory mediator production and its release into the circulation (9), alongside activation of various receptors within the lung (Toll-like receptor 4 [TLR-4], receptor for advanced glycation end products [RAGEs]) by damage-associated molecular pattern molecules (DAMPs) (10, 11), may drive the development of pulmonary injury through increased mediator production and infiltration of activated neutrophils (7, 12, 13).

In this study, we examined the effects of mild TBI on pulmonary neutrophil priming. To our knowledge, this has never been investigated because most researchers have studied the pulmonary response to moderate/severe TBI models. We show for the first time that mild TBI induces massive pulmonary sequestration of interstitial neutrophils that then transmigrate into the alveolar compartment following a subsequent small inflammatory insult such as acid aspiration to induce injury. This has potential implications for considering earlier prophylactic measures for targeting neutrophil migration from the interstitium to the alveolar space in patients with TBI.

## METHODS

### Animals

Eight-week-old male CD1 mice were purchased from Harlan (Harlan, United Kingdom) and given 1 week to acclimatize before experimentation. Mice were maintained in 12-hour light/12-hour dark cycles with free access to food and water. All experimental animal procedures were approved by the University of Edinburgh and were performed in accordance with Home Office guidelines (Animal [Scientific Procedures] Act 1986).

### Fluid Percussion Injury

Adult (25–35 g) male CD1 mice were anesthetized with isoflurane (Merial, Woking, United Kingdom) and prepared for a 1.5 atmosphere fluid percussion injury (FPI) according to published methodology (14). For full details and flow charts outlining experimental protocols, see **online data supplement** (Supplemental Digital Content 1, http://links.lww.com/CCM/D740) and **Supplementary Figure E1** (Supplemental Digital Content 2, http://links.lww.com/CCM/D741; **legend**, Supplemental Digital Content 1, http://links.lww.com/CCM/D740). Animals (*n* = 3–6 per group) were retrieved at 0, 6, 24, and 48 hours post-FPI.

### Brain Histology and Immunohistochemistry Preparation

For histologic and immunohistochemical assessment of neuronal damage and cellular infiltration, brains were frozen in −38°C isopentane (277258-1L; Sigma-Aldrich Company, St. Louis, MO) before being placed in storage at −80°C. Tenmicrometer coronal cryostat sections were cut using the Leica CM1900 (Leica, Wetzlar, Germany) before being mounted on Leica Surgipath X-tra Adhesive (3800050, Leica) precleaned micro slides and stored at −80°C. Light microscopy images were obtained using a Zeiss Axioskop (Carl Zeiss, Welwyn Garden City, United Kingdom) light microscope connected to a Qimaging Micropublisher 3.3 camera (Qimaging, Surrey, BC, Canada).

### Acid Fuchsin

To assess neuronal damage, brain sections were stained with acid fuchsin. Frozen 10-µm coronal cryostat sections were dried at 40°C overnight before being fixed in cold 4% paraformaldehyde in PBS for 1 hour. Slides were washed in phosphate-buffered saline (3 × 5 min) before being stained for 30 seconds in 1% Acid Fuchsin (A3908; Sigma-Aldrich Company, St. Louis, MO) with three drops per 100 mL of glacial acetic acid. Slides were washed with water, dehydrated through 70%, 90%, and 100% ethanol for 2 minutes each, and cleared in xylene for 2 minutes before being mounted with Pertex (3808707E; Leica, Wetzlar, Germany). To quantify neuronal damage, three sections located throughout the hippocampus of each animal were imaged, with acid fuchsin positive cells counted using Image J (National Institutes of Health, Bethesda, MD).

### Myeloperoxidase Immunohistochemistry

To assess neutrophil infiltration, myeloperoxidase immunohistochemistry was performed. For full details, see online data supplement (Supplemental Digital Content 1, http://links.lww.com/CCM/D740).

### Flow Cytometric Analysis of Brain and Lung Tissues

Tissue digests and flow cytometry methods are detailed in the online data supplement (Supplemental Digital Content 1, http://links.lww.com/CCM/D740).

### Bronchoalveolar Lavage

Bronchoalveolar lavage fluid (BALF) was collected according to a published protocol (15). For full details, see online supplement (Supplemental Digital Content 1, http://links.lww.com/CCM/D740).

### Enzyme-Linked Immunosorbent Assay

Enzyme-linked immunosorbent assay (ELISA) kit for the measurement of interleukin (IL)-1β in BALFs (DuoSet; R&D Systems, Minneapolis, MN) was used according to the manufacturers’ instructions.

### Total Protein

Total protein within BALF was performed using a Pierce BCA Total Protein Assay Kit (23227; Thermo Scientific, Waltham, MA) as per the manufacturers’ instructions.

### Hydrochloric Acid Microaspiration Model

To model acid microaspiration following TBI, a subclinical hydrochloric acid (HCl) aspiration model was developed (16, 17). HCl (318965; Sigma-Aldrich Company, St. Louis, MO) was diluted to pH 1.75 in saline (0.9% sodium chloride, UKF7124; Baxter, Deerfield, IL), and 50 µL was administered via an “intratracheal” route. 11.25 µl of HCl was instilled in a volume of 50 µL. An equivalent scaling factor in humans for lung weight and size is approximately 700-fold; therefore, the equivalent volume in a human would be approximately 7 mL. After receiving HCl, a preemptive intraperitoneal injection of saline (200 µL) was given. Mice were then placed in a warm humidified oxygen chamber (2 L/min flow rate) for 30 minutes before reversal of anesthesia. Mice were kept on a heat mat before retrieval 6 hours later. To model acid microaspiration following TBI, the HCl model was applied immediately following FPI after recording the righting time.

### Neutrophil Depletion—Anti-Lymphocyte Antigen 6 Complex Locus G6D Antibody

Neutrophil depletion was achieved with an anti-lymphocyte antigen 6 complex locus G6D (LY-6G) monoclonal antibody (clone IA8; BioXCell, West Lebanon, NH) as described (15). For details of flow cytometric analysis of blood, see online data supplement, (Supplemental Digital Content 1, http://links.lww.com/CCM/D740).

### Cytokine Bead Array

Cytokine levels within BALF were measured using the BD Cytometric Bead Array Mouse Inflammation Kit (552364; BD Biosciences, San Jose, CA) as per the manufacturers’ instructions.

### Statistics

Data are represented as mean ± sd or sem. Quantification of histology/immunohistochemistry was performed blinded by the investigator. Statistical comparisons were made using two-tailed Student *t* test or one-way/two-way analysis of variance with Bonferroni posttest for multiple comparisons. A *p* value of less than 0.05 was considered statistically significant (*p < 0.05, **p < 0.01, ***p < 0.001). All graphs and statistics were performed using the statistical package Graphpad Prism 5 for Windows (Graphpad Software, La Jolla, CA).

## RESULTS

### A 1.5 ATM FPI in Mice Induces a Unilateral Cortical Insult With Cerebral Inflammatory Cell Influx and No Measureable Clinical Sequelae

**Figure 1.**
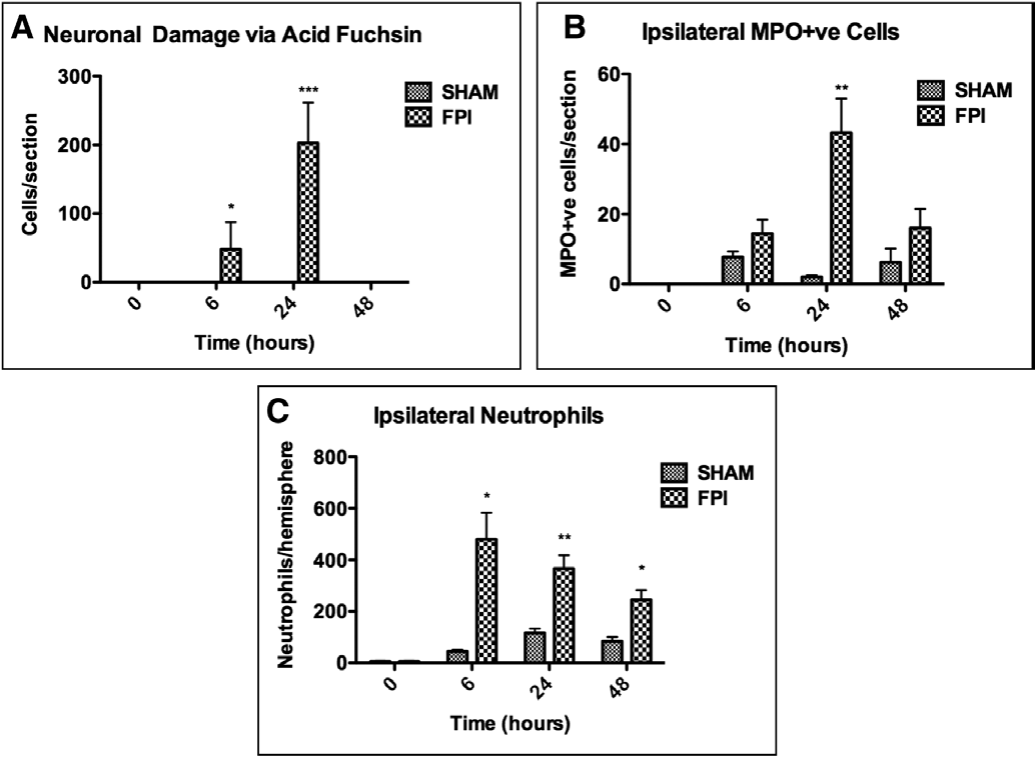
Characterization of mild fluid percussion-induced brain injury. Individual hemispheres were digested 6, 24, and 48 hr after receiving fluid percussion injury (FPI)/sham procedures for flow cytometry or processed for histology. **A**, Neuronal damage via acid fuchsin. Significant neuronal damage was detected in the ipsilateral hemisphere 6 and 24 hr after receiving FPI. **B**, Myeloperoxidase (MPO) immunohistochemistry on brain sections 24 hr after sham/FPI procedure. Significant neutrophil infiltration was seen at 24 hr after FPI. **C**, Ipsilateral neutrophil quantification via flow cytometry. A small number of neutrophils were detected in the ipsilateral hemisphere 6, 24, and 48 hr after FPI. Data are represented as mean ± sem or sd (Fig. 1*C*) and were analyzed using two-way analysis of variance (*n* = 3–5 per group, *p < 0.05, **p < 0.01, ***p < 0.001).

Mice received a lateral FPI using an established methodology (14). Sham and FPI were significantly different in righting times (time to turn prone from supine), indicating that a significant cortical impact had occurred (**Supplementary Fig. E2**, Supplemental Digital Content 3, http://links.lww.com/CCM/D742; **legend**, Supplemental Digital Content 1, http://links.lww.com/CCM/D740). However, once the mice had recovered after the impact (within 15 min), no behavioral or physical differences could be distinguished between the two groups. To assess neuronal injury, brain sections were stained with acid fuchsin. Homogenous staining and cells with regular morphology were seen following sham treatment, indicating no neuronal damage (for all histology and flow cytometry plots, see **Supplementary Fig. E3**, Supplemental Digital Content 4, http://links.lww.com/CCM/D743; legend, Supplemental Digital Content 1, http://links.lww.com/CCM/D740). However, significant neuronal damage, evident by the appearance of triangularly shaped, intensely stained neurons with condensed cellular morphology and pyknotic nuclei, could be seen 6 and 24 hours after FPI (**Fig. 1*A***). Damage was located to the injury site and the parietal cortex within the ipsilateral hemisphere. No damage was evident within the contralateral hemisphere or within the corpus callosum or hippocampus following FPI or sham procedures.

Neuronal injury following FPI was associated with significant neutrophil infiltration in the ipsilateral hemisphere adjacent to the injury/craniotomy site compared with sham treatment (**Fig. 1*B***). This was confirmed using flow cytometry, which identified significant yet mild infiltration into the ipsilateral (injured) hemisphere following FPI at all time points (**Fig. 1*C***). The temporal quantification of infiltration differed slightly to that seen with immunohistochemistry, most likely due to the increased sensitivity of flow cytometry and its ability to count neutrophils that were extravasating from blood vessels. When healthy, untouched brains were digested, no neutrophils were detected.

### Mild Fluid Percussion Cortical Injury Induces Massive Pulmonary Interstitial Neutrophil Migration Without Pulmonary Vascular Leak

Lungs from FPI and sham animals were assessed for evidence of inflammation. Both the alveolar and interstitial compartments were evaluated at 0, 6, 24, and 48 hours after FPI. In BALF, only resident alveolar macrophages were present from sham- or FPI-treated animals (**Supplementary Fig. E4**, Supplemental Digital Content 5, http://links.lww.com/CCM/D744; legend, Supplemental Digital Content 1, http://links.lww.com/CCM/D740). No differences in BALF cell counts, alveolar IL-1β levels, or vascular permeability were observed between FPI- and sham-treated animals (**Supplementary Fig. E5**, Supplemental Digital Content 6, http://links.lww.com/CCM/D745; legend, Supplemental Digital Content 1, http://links.lww.com/CCM/D740).

**Figure 2.**
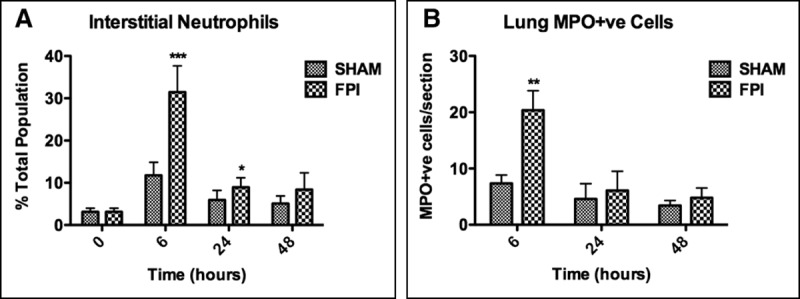
Pulmonary interstitial infiltration of neutrophils following concussion fluid percussion injury (FPI). Interstitial lung tissue was collected 6, 24, and 48 hr after FPI/sham procedure to assess signs of pulmonary injury/inflammation. **A**, Interstitial neutrophils. Significant neutrophil accumulation was present 6 and 24 hr after FPI procedure. **B**, Myeloperoxidase (MPO) immunohistochemistry on lung sections following sham/FPI procedure. Significantly more neutrophils were seen 6 hr after FPI, which confirmed the findings seen with flow cytometry. Data are represented as mean ± sd or sem (**B**) and were analyzed using two-way analysis of variance (*n* = 3–6 per group, *p < 0.05, ***p < 0.001).

To quantify pulmonary interstitial myeloid cell accumulation, whole lungs were digested after perfusion and lavage for multiparametric flow cytometric analysis as previously described (17). Significant neutrophil accumulation occurred within the interstitium 6 and 24 hours after FPI compared with sham treatment (**Fig. 2*A***; and **Supplementary Fig. E6**, Supplemental Digital Content 7, http://links.lww.com/CCM/D74; legend, Supplemental Digital Content 1, http://links.lww.com/CCM/D740). This was confirmed using immunohistochemistry for myeloperoxidase (**Fig. 2*B***). To investigate one potential mechanism driving high interstitial neutrophil accumulation, the intercellular adhesion molecule (ICAM)-1 expression of circulating and pulmonary neutrophils was assessed. No detectable changes in ICAM-1 expression were identified (**Supplementary Fig. E7**, Supplemental Digital Content 8, http://links.lww.com/CCM/D747; legend, Supplemental Digital Content 1, http://links.lww.com/CCM/D740). BALF was also analyzed on a proteome profiler array to characterize cytokine expression. Pooled samples from three mice per group were assessed. FPI was shown to increase the expression of 105 proteins, including neutrophil chemokines chemokine (C-C motif) ligand 2 (CCL2)/monocyte chemotactic protein (MCP)-1, chemokine (C-X-C motif) ligand 2 (CXCL2)/macrophage inflammatory protein (MIP)-2, IL-6, and tumor necrosis factor (TNF), all of which were absent following sham treatment, as well as an increase in RAGE expression (**Supplementary Fig. E8**, Supplemental Digital Content 9, http://links.lww.com/CCM/D748; legend, Supplemental Digital Content 1, http://links.lww.com/CCM/D740; and **Supplementary Table E1**, Supplemental Digital Content 1, http://links.lww.com/CCM/D740).

### Intratracheal Acid Aspiration After FPI Results in Alveolar Neutrophilia and Pulmonary Hemorrhage

The observation that mild concussive FPI results in massive interstitial neutrophil accumulation without alveolar neutrophil ingress suggested that the intra-alveolar compartment may be primed for subsequent intrapulmonary insult. To test this, we established a “double hit” model in which mice received a small volume of HCl, which in naive mice caused no lung injury, directly into the lungs immediately after FPI or sham injury.

**Figure 3.**
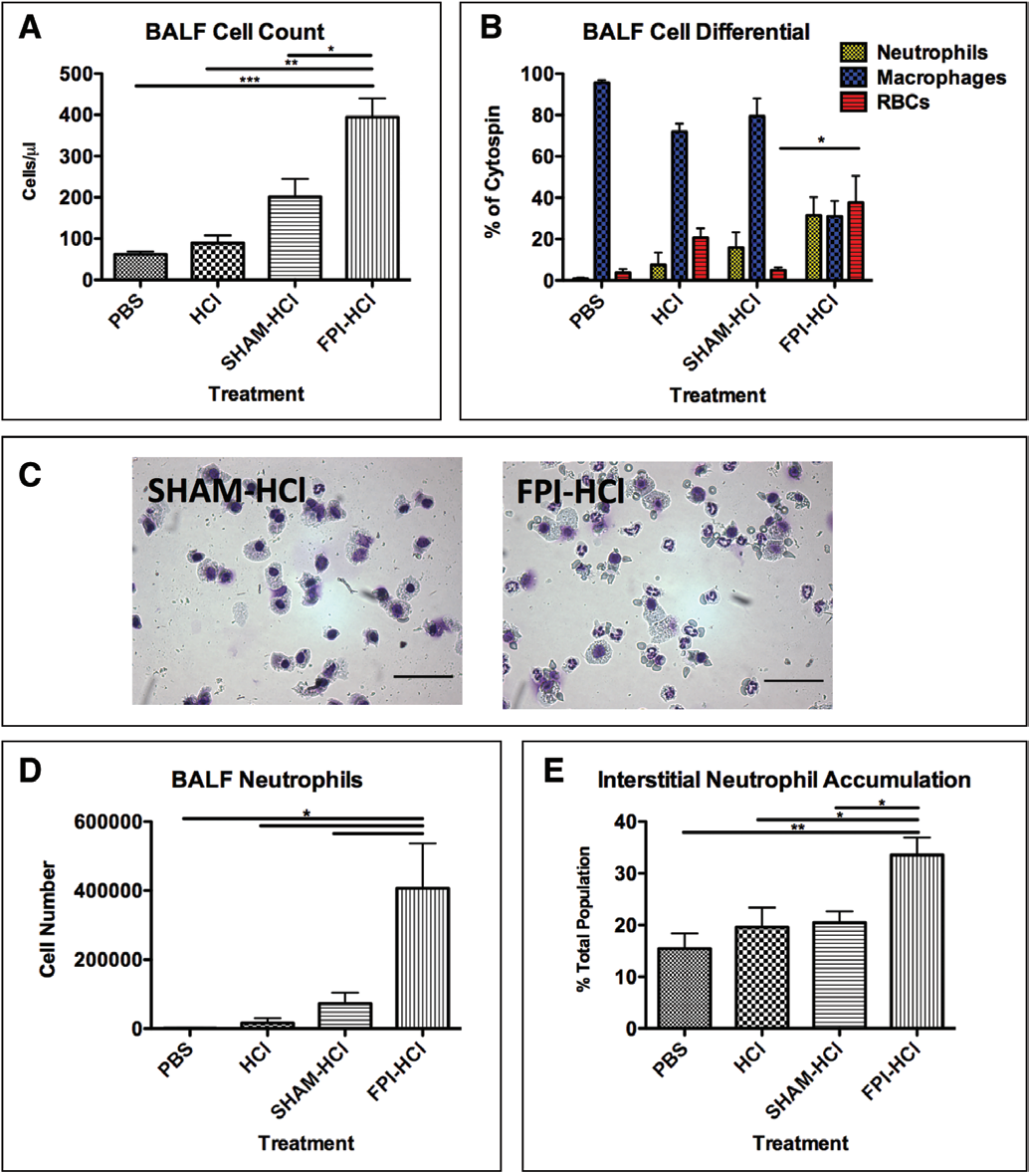
Bronchoalveolar lavage fluid (BALF) and interstitial analysis following fluid percussion injury (FPI) and intratracheal hydrochloric acid (HCl). Fifty-microliter pH 1.75 HCl was instilled via an intratracheal route immediately after FPI/sham procedure and lungs retrieved 6 hr later. **A**, FPI-treated mice had significantly higher BALF cell counts 6 hr after receiving HCl compared with controls. **B**, BALF cell differential. HCl administration after both FPI and sham treatments resulted in neutrophil influx; however, FPI-treated mice had significant pulmonary hemorrhage. **C**, Representative sham-HCl and FPI-HCl cytocentrifuge preparations. Neutrophils and resident alveolar macrophage are the predominant cells present within sham-HCl BALF. RBCs, however, were also present following FPI-HCl. **D**, BALF neutrophil count. FPI-HCl–treated mice had significantly more neutrophils within the BALF than in controls. **E**, Interstitial neutrophil accumulation. Significant neutrophil accumulation was seen following FPI-HCl. Data are represented as mean ± sd and were analyzed using one-way analysis of variance (*n* = 4 per group, *p < 0.05). Scale bar represents 100 μm.PBS = phosphate-buffered saline.

In naive mice, intratracheal HCl (pH, 1.75) induced no increase in BALF cell counts when compared with PBS (**Fig. 3*A***). However, after mild concussive FPI, HCl administration significantly increased alveolar cell counts. This was associated with significant pulmonary hemorrhage (**Fig. 3**, ***B*** and ***C***). Quantification of neutrophils confirmed significant alveolar infiltration in FPI-HCl–treated mice compared with sham-HCl (**Fig. 3*D***). Nonsignificant neutrophil levels were detected within the pulmonary interstitium following HCl administration alone or in sham-HCl mice; however, levels were significantly increased following FPI-HCl (**Fig. 3*E***).

### Neutrophil Depletion With Anti-LY-6G Depleting Antibody Attenuates FPI-HCl Injury

**Figure 4.**
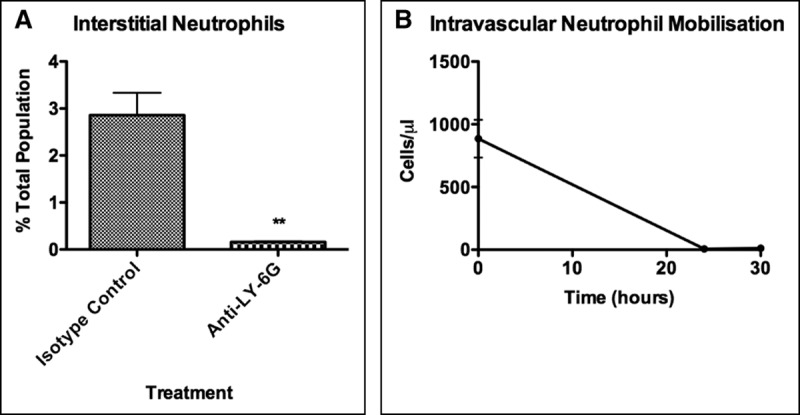
Confirmation of neutrophil depletion with anti-lymphocyte antigen 6 complex locus G6D (LY-6G). Mice were preinjected with anti-LY-6G 24 hr before receiving fluid percussion injury. **A**, Pulmonary interstitial neutrophils. Neutrophils could still be identified following isotype control; however, anti-LY-6G successfully depleted neutrophils within the lung. **B**, Intravascular neutrophil mobilization. Anti-LY-6G injection resulted in total depletion of circulating neutrophils throughout the experiment. Data are represented as mean ± sd and were analyzed using Student *t* test and one-way analysis of variance (*n* = 3–6 per group, *p < 0.05, **p < 0.01, ***p < 0.001).

To determine if neutrophils had a direct association with alveolar pulmonary hemorrhage, neutrophil depletion studies were performed using an anti-LY-6G depleting antibody as described (15). Time course experiments confirmed that 24 hours after injection, anti-LY-6G–depleting antibody successfully depleted neutrophils within the pulmonary interstitium (**Fig. 4*A***; and **Supplementary Fig. E9**, Supplemental Digital Content 10, http://links.lww.com/CCM/D749; legend, Supplemental Digital Content 1, http://links.lww.com/CCM/D740). Total depletion of circulating neutrophils was confirmed throughout the duration of the double hit experiment (**Fig. 4*B***).

**Figure 5.**
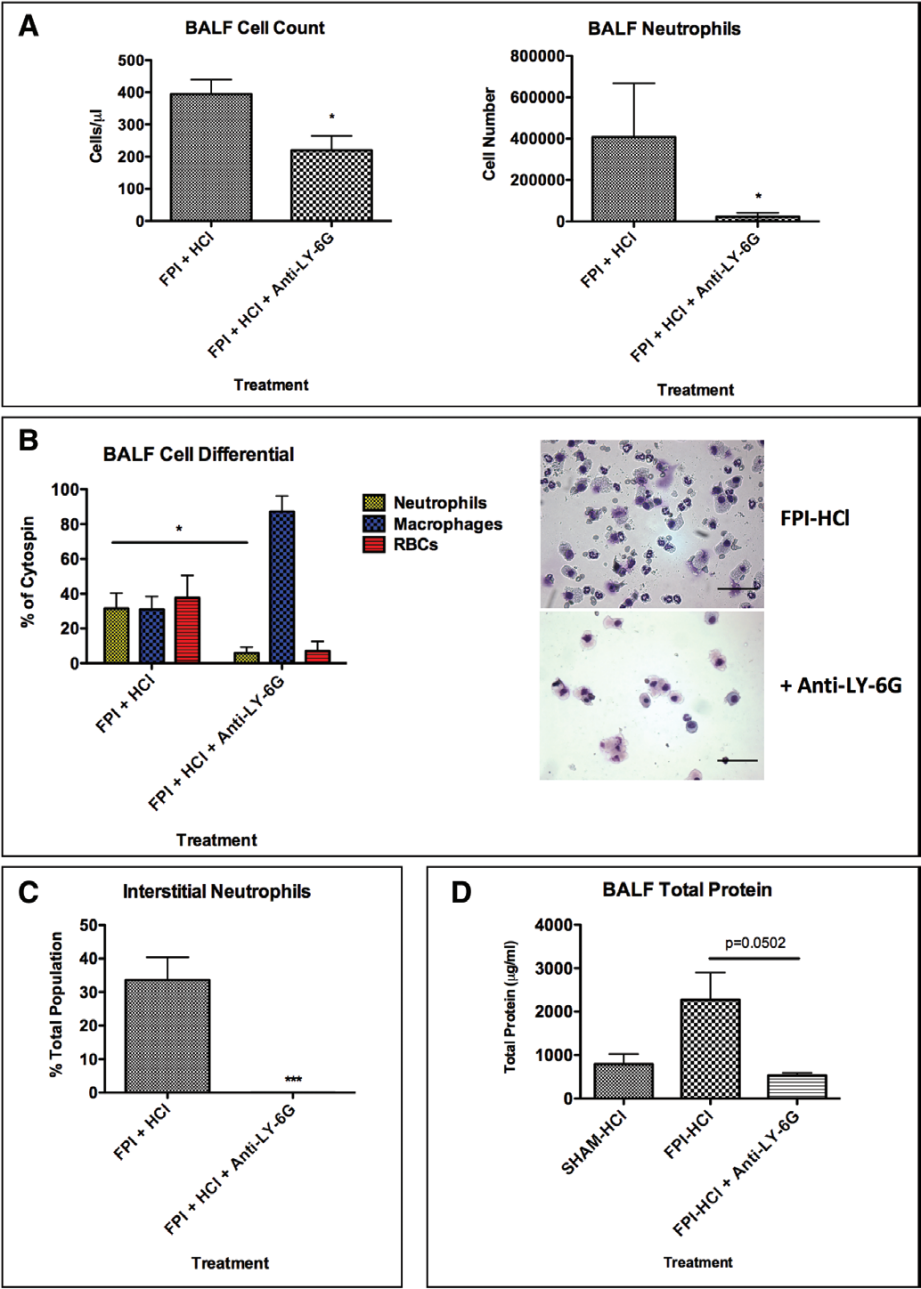
Hydrochloric acid (HCl) double hit at righting time with anti-lymphocyte antigen 6 complex locus G6D (LY-6G). Mice were preinjected with anti-LY-6G 24 hr before receiving fluid percussion injury (FPI)-HCl. Bronchoalveolar lavage fluid (BALF) was retrieved 6 hr after FPI. **A**, BALF analysis. Administration of anti-LY-6G resulted in a significant drop in BALF cell count due to a reduction in BALF neutrophils. **B**, BALF cell differential and representative cytocentrifuge preparations of FPI-HCl and FPI-HCl + anti-LY-6G. Anti-LY-6G resulted in a significant drop in BALF neutrophils and a nonsignificant reduction in pulmonary hemorrhage. **C**, Interstitial neutrophil accumulation. Administration of anti-LY-6G resulted in a significant reduction in interstitial neutrophil accumulation. **D**, BALF total protein. An increase in vascular permeability was observed in FPI-HCl–treated mice compared with sham-HCl. Neutrophil depletion resulted in a reduction in vascular permeability. Data are represented as mean ± sd and were analyzed using Student *t* test and one-way analysis of variance (*n* = 3–6 per group, *p < 0.05, **p < 0.01, ***p < 0.001). Scale bar represents 100 μm.

A significant reduction in BALF cell counts was seen in the FPI-HCl double hit following neutrophil depletion (**Fig. 5*A***). BALF macrophage levels remained unchanged (**Supplementary Fig. E10**, Supplemental Digital Content 11, http://links.lww.com/CCM/D750). This reduction in neutrophil number was associated with a decrease in pulmonary hemorrhage (**Fig. 5*B***). Addition of anti-LY-6G–depleting antibody resulted in significant depletion of interstitial neutrophils (**Fig. 5*C***).

The increase in vascular permeability observed in FPI-HCl–treated mice was prevented following neutrophil depletion (**Fig. 5*D***). When inflammatory cytokine levels were assessed, a trend of increased TNF, MCP-1, and IL-6 was seen in FPI-HCl–treated mice compared with sham-HCl (Supplementary Fig. E10, Supplemental Digital Content 11, http://links.lww.com/CCM/D750; legend, Supplemental Digital Content 1, http://links.lww.com/CCM/D740). Neutrophil depletion resulted in a reduction in TNF and IL-6 levels, however, had no effect on MCP-1 levels.

## DISCUSSION

Lung injury following TBI is a common and serious consequence. Understanding the pathophysiologic mechanisms that underlie the development of lung injury after TBI may help identify novel therapeutic avenues because current treatment interventions are limited. The brain-lung axis has been postulated to involve complex reciprocal cross talk (18), and pulmonary dysfunction is a predictor of poor neurologic outcome and mortality (3). Hence, it is now well recognized that ventilatory strategies after brain injury should include lung-protective approaches (19), which also have the potential to increase the number of eligible lungs for donation (20). We show for the first time that mild TBI increases pulmonary susceptibility to a secondary innocuous insult such as miniscule acid microaspiration. Even more importantly, we identify pulmonary interstitial neutrophils as the key cells responsible for driving augmented injury.

To explore the brain-lung axis, we hypothesized that subclinical pulmonary priming may contribute to subsequent lung injury. To test this, we developed an experimental model of TBI and subsequently delivered acid to the lung. An injury force of 1.5 ATM was chosen because this was expected to produce a moderate yet fully clinically reversible phenotype mimicking a concussion (21). To ensure sufficient cortical injury, the righting times for mice to recover following FPI/sham procedure were recorded as previous studies in rats have shown correlation between length of unconsciousness and degree of pathologic consequences (22). An exclusion time of 270 seconds was chosen (23); hence, any animal receiving FPI with a righting time less than 270 seconds was removed from the study. At an injury level of 1.5 ATM, righting times were well above 270 seconds, no mortality was seen and no immediate or long-term clinical sequelae were observed once the mice began to mobilize.

To characterize the cerebral injury, brains were sectioned and stained with acid fuchsin, an anionic dye that stains acidophilic irreversibly damaged neurons a dark red (24, 25). Damage was observed directly below the injury site, resulting from the fluid pulse. By 24 hours, neuronal damage had increased in size, suggesting secondary injury. This was associated with significant neutrophil infiltration 6, 24, and 48 hours after FPI in the ipsilateral hemisphere. Neutrophils, capable of secreting neurotoxic substances (26), exacerbate excitotoxic insults and increase neuronal death in vitro (27). DAMPs (11) and proinflammatory cytokines (IL-1β, IL-6, and TNF) (28) released from the injured brain attract inflammatory cells to contused cerebral tissue.

The extent of neuronal damage (confined only to cortex and small cell number), small degree of neutrophil infiltration (< 500 cells), and lack of any overt clinical signs between FPI and sham groups demonstrated that this injury was more representative of mild “concussive” TBI, even though 1.5 ATM has been previously considered within the moderate severity range in mice (21).

BALF differential cell counts showed no differences in alveolar cell count between FPI- and sham-treated animals at any time point. Alveolar neutrophil infiltration was absent, and no differences were observed in vascular permeability.

Surprisingly, mild TBI induced extensive interstitial neutrophil accumulation, which remained elevated 24 hours after FPI. There were no observable differences in pulmonary vascular permeability, suggesting that a sympathetic catecholamine storm was not responsible for driving high interstitial neutrophil accumulation in this model as endothelial dysfunction is a recognized major component of sympathetic catecholamine storms. TBI has previously been shown to increase levels of pulmonary IL-1β, IL-6 (13), prostaglandin-synthesizing enzyme cyclooxygenase-2 (5), and leukotriene B_4_; the latter being particularly chemotactic for neutrophils (13). Despite no differences in IL-1β being seen in this model at 6 hours post-FPI, increased expression of other direct or indirect neutrophil chemoattractants were identified using the proteome profiler array (CCL2/MCP-1, CXCL1/KC, CXCL2/MIP-2, IL-6, TNF) (13, 29–31). RAGE was also up-regulated following FPI. This receptor has been implicated with the development of pulmonary inflammation following TBI through its interaction with high-mobility group box-1, a DAMP released from the injured brain that leads to sustained activation of nuclear factor κ-light-chain-enhancer of activated B cells (leading to proinflammatory cytokine production) and increased RAGE expression to ensure amplification of the inflammatory signal (11, 32). TLR-4 activation by DAMPs may also account for the increase in proinflammatory cytokines (10).

Seeing as no differences in ICAM-1 expression were detected in circulating and pulmonary neutrophils, reverse transmigration of neutrophils from the injured brain to the lung was ruled out (33).

To investigate whether mild TBI increased susceptibility to pulmonary injury, an acid microaspiration “double hit” model was established. Acid microaspiration is a common sequelae in patients who are concussed or lose airway protection as damage to the CNS has been associated with an increased risk of stomach content microaspiration (4). To model acid microaspiration following TBI, HCl (pH 1.75 in sodium chloride resembling the acidity and osmolality similar to that of patient gastric sections) (34) was administered via an intratracheal route immediately following FPI (after recording the righting time). Pulmonary consequences were evaluated 6 hours later.

As expected, HCl administration in naive mice was innocuous due to the large size of mice (25–35 g) and high pH when compared with previous work (16). HCl following FPI, however, resulted in a significant increase in alveolar neutrophil infiltration which was not seen in sham-HCl mice. This was accompanied with increases in the proinflammatory cytokines TNF, MCP-1, and IL-6 and significant interstitial neutrophil accumulation. Alveolar and interstitial neutrophil accumulation were associated with pulmonary hemorrhage and increased vascular permeability. Neutrophil-specific depletion with an anti-LY-6G antibody confirmed neutrophil dependence of vascular leak.

Neutrophil-mediated lung injury is central to the pathogenesis of many lung conditions including ARDS (17, 35, 36), and the data presented here suggest that even in mild experimental TBI, neutrophilic interstitial infiltration is an important prelude to subsequent innocuous intrapulmonary microaspiration. Although the mechanisms remain to be delineated, inhibition or attenuation of neutrophil recruitment/activity may be a potential therapeutic intervention following TBI. These data suggest that TBI patients with reduced consciousness may be susceptible to the immediate effects of microaspiration or indeed to other injurious iatrogenic insults such as ventilation-induced stretch that could direct the intra-alveolar recruitment of neutrophils from the interstitial pool.

The findings in this article support the underlying hypothesis that the brain-lung axis may contribute to priming for secondary neutrophil-dependent lung injury. To investigate mechanisms of neutrophil recruitment, further experiments include blocking key neutrophil pathways that may contribute to recruitment and activation such as phosphatidylinositide 3-kinase and inhibitors of early alarm cytokines such as TNF. Additionally, augmenting neutrophil clearance through promoting apoptosis is also a viable experimental intervention (37). Early administration of glucocorticoids may also help reduce inflammation; however, care must be given to the dosage and timing because glucocorticoids also promote neutrophil survival, thus potentially prolonging inflammation.

Furthermore, in the clinical setting, it will be necessary to prove whether these observations also prevail after minor TBI. Human experimental studies are now warranted to quantify the accumulation of neutrophils in the lung post-TBI through human molecular imaging approaches including both whole-body approaches such as positron emission tomography (38) and evolving endomicroscopic approaches that have the potential to visualize neutrophil accumulation at the bedside in critical care (39). These studies would enable targeted therapeutic intervention.

## ACKNOWLEDGMENTS

We thank the Queen’s Medical Research Institute Flow Cytometry and Histology Facilities at the University of Edinburgh for assistance.

## Supplementary Material
