# Supplementary material for: Cerebral Concussion Primes the Lungs for Subsequent Neutrophil-Mediated Injury
Source: Crit Care Med. 2018 Aug 15;46(9):e937–44. doi: 10.1097/CCM.0000000000003270 (PMC6110623; doi:10.1097/CCM.0000000000003270)
Supplement: Supplementary file 1 [file ccm-46-e937-s001.docx]

**Online Data Supplement**

**Cerebral concussion primes the lungs for subsequent neutrophil-mediated injury**

Duncan C. Humphries, Stephen O’Neill, Emma Scholefield, David A. Dorward, Alison C. Mackinnon, Adriano G. Rossi, Christopher Haslett, Peter J.D. Andrews, Jonathan Rhodes & Kevin Dhaliwal

**Additional Methods**

**Fluid Percussion Injury**

Adult (25-35g) male CD1 mice were anaesthetized with isoflurane (Merial, UK) and positioned in a stereotaxic frame (Model 900, David Kopf Instruments, Tujunga, California, USA) where anaesthesia was maintained via a nose cone. Body temperature was maintained at 37±0.5⁰C using a heating pad unit and rectal thermometer probe (Homoeothermic blanket control unit, Harvard Apparatus Ltd, Edenbridge, UK). Animals were prepared for fluid percussion according to the method described (1). Briefly, a midline incision was performed to expose the skull and a 2.0mm diameter right-sided parasagittal craniotomy was trephined midway between bregma and lambda sutures, 2mm from the sagittal suture. An injury cannula (adapted from a 20-gauge hypodermic needle with a Luer-Lock fitting) was then secured around the craniotomy using cyanoacrylate glue and dental cement (Simplex Red, Associated Dental Products, Swindon, UK). Animals were pre-oxygenated with 100% oxygen and connected to the FPI device (Custom Design and Fabrication, Virginia Commonwealth University, USA). At the 1^st^ sign of a response from a paw-pinch, mice received a 1.5ATM fluid percussion injury. Sham animals underwent identical procedures however did not receive an FPI as the pendulum was not released. Righting times were then measured and an exclusion time of 270 seconds was set to ensure each animal received sufficient injury, with animals righting themselves within this time period being removed from the study. Following FPI, the injury cannula was removed and injury site inspected for dural integrity. The scalp wound was sutured and Bupivacaine, a local anaesthetic, was administered (2mg/kg, AstraZeneca UK Ltd, Luton, UK). After recovering in a heated incubator (Animal intensive unit, Lyon Electric Company, Los Angeles, California, USA), mice were returned to their cages.

**Brain Myeloperoxidase Immunohistochemistry**

Frozen 10μm coronal cryostat sections were allowed to dry at 40°C overnight before being fixed in cold 4% Paraformaldehyde in PBS for 30 seconds. Slides were then washed in PBS (3 x 5 minutes) before being dipped in -20°C acetone for 5 minutes. This was again followed by another wash in PBS (3 x 5 minutes). Endogenous peroxidase activity was quenched using 3% hydrogen peroxide (H1009, Sigma- Aldrich Company Ltd) in methanol for 5 minutes and then rinsed in PBS (3 x 5 minutes). Normal Goat Serum (NGS) in PBS (200μL in 10mL) was then used for 1 hour at room temperature to block unspecific binding. A polyclonal rabbit anti- mouse myeloperoxidase antibody (ab9535, Abcam, Cambridge, UK) was diluted 1:50 in NGS/PBS and added to each slide overnight at 4°C before being rinsed in PBS (3 x 5 minutes). For a negative control, primary antibody was excluded from sections. The goat anti-rabbit IgG Vectastain ABC kit (PK-6100, Vectastain Laboratories Ltd, Peterborough, UK) was then used for antigen visualization according to the manufacturer’s instructions. The biotinylated antibody (E0432, Dako, Cambridge, UK) was diluted 1:200 in NGS/PBS (150μL in 10mL) and added for 1 hour at room temperature. Slides were then washed with PBS (3 x 5 minutes) prior to the addition of the avidin/biotinylated peroxidase enzyme complex (ABC) for 30 minutes. After a subsequent wash with PBS (3 x 5 minutes), 3,3’-diaminobenzidine (DAB, SK-4100, Vector Laboratories Ltd) was added to visualize antigen staining. This reaction was terminated with water before being counterstained with Harris’ haematoxylin (3801560BBE, Leica, Milton Keynes, UK) for 30 seconds. Slides were then washed with water, dehydrated through 70, 80, 95 and 100% ethanol for 20 seconds each and cleared in xylene for 10 minutes before being mounted with Pertex (3808707E, Leica, Milton Keynes, UK). To quantify MPO+ve cells within the brain, 3 sections located throughout the hippocampus of each animal were imaged, with MPO+ve cells (DAB+ve) counted using Image J (National Institutes of Health, USA).

**Lung Myeloperoxidase Immunohistochemistry**

Following exsanguination and retrieval of BALF, lungs were perfused with 10mL PBS through the right ventricle. The lungs were removed and inflated using Methacarn (60% absolute methanol, 30% chloroform, 10% glacial acetic acid – all chemicals sourced from Sigma- Aldrich Company Ltd) via the trachea, and stored for 24 hours in Methacarn before being transferred to 70% ethanol. Lungs were then paraffin-wax embedded prior to being sectioned by the University of Edinburgh’s Histology department (Queens Medical Research Institute, Little France, University of Edinburgh).

To enable immunohistochemistry, sections were first de-waxed using Xylene (2 x 5 minutes), and then re-hydrated through 100, 95, 80 and 70% ethanol for 20 seconds each before being placed in PBS. Sections were then permeabilised using Triton-X- 100 (Sigma-Aldrich Company Ltd) for 10 minutes and washed with PBS (3 x 5 minutes). Endogenous peroxidase activity was then quenched using 3% hydrogen peroxide (H1009, Sigma-Aldrich Company Ltd) for 5 minutes, before being washed again with PBS (3 x 5 minutes). Blocking was then performed using normal goat serum (NGS) in PBS (200μL in 10mL) for 1 hour at room temperature. A polyclonal rabbit anti-mouse myeloperoxidase antibody (ab9535, Abcam, Cambridge, UK) was diluted 1:100 in NGS/PBS and added to each slide. As a negative control, the primary antibody was excluded from some sections. Slides were incubated overnight at 4⁰C prior to another wash with PBS (3 x 5 minutes). Antigen visualization was performed using the Goat Anti-Rabbit Vectastain Elite ABC kit (PK-6100, Vector Laboratories Ltd, Peterborough, UK) according to manufacturer’s instructions. The biotinylated antibody (E0432 Dako, Cambridge, UK) was diluted 1:200 in NGS/PBS (150μL in 10mL) and applied for 1 hour at room temperature. Following another wash with PBS (3 x 5 minutes), the avidin/biotinylated peroxidase enzyme complex (ABC) was added for 30 minutes at room temperature. Sections were again washed with PBS (3 x 5 minutes) and antigen staining visualization performed with 3-3’- Diaminobenzidine (DAB, SK-4100, Vector Laboratories Ltd) for 5 minutes. The colour reaction was terminated with water, with sections counterstained in Harris’ Haematoxylin (3801560BBE, Leica, Milton Keynes, UK) for 1 minute prior to dehydration through 70, 80, 95 and 100% ethanol for 20 seconds each. Slides were then cleared in Xylene (2 x 5 minutes) and mounted with Pertex (3808707E, Leica, Milton Keynes, UK).

To quantify MPO +ve cells within the lung, 5 fields were selected at random from each animal and imaged, with MPO +ve cells (DAB +ve) counted using Image J (National Institutes of Health, USA).

**Bronchoalveolar Lavage**

Bronchoalveolar lavage fluid (BALF) was obtained by exposing the trachea and inserting a plastic-coated 25-gauge needle which was secured in place with elasticated thread. 3 boluses of 0.8mL PBS were then instilled and retrieved prior to being stored on ice. The first bolus was kept separate from the subsequent two. Lavages were weighed to establish total volume, before being centrifuged at 350g for 5 minutes. Supernatants were removed and stored at -80⁰C prior to cytokine/protein analysis. Cell pellets were then combined in 1mL PBS prior to performing a differential cell count on cytocentrifuged preparations stained with Quick-Diff kit (102164, Reagena, West Sussex, UK).

**Flow Cytometric Brain Digest**

For brain digestion and subsequent flow cytometric analysis, male CD1 mice were deeply anaesthetized with sodium pentobarbital (Euthatal, Merial, UK) *i.p*. To remove non-adherent cells, 10mL of PBS was flushed through the beating left ventricle after cutting the inferior vena cava to allow exsanguination. Brains were quickly dissected free and placed into 2mL of cold RPMI 1640 medium. After removing the supernatant, individual cerebral hemispheres were placed in a 1:3 DNase:collagenase mix (DNase 1, 10mg/mL, DN-25, Sigma-Aldrich Company Ltd; Collagenase D, 10mg/mL, 11088866001, Roche, Basel, Switzerland) before being disrupted with scissors. Cells were further released from tissue via vigorous pipetting and incubated at 37⁰C for 40 minutes. Cells were centrifuged at 350g for 5 minutes and resuspended in a 30% Percoll (17089101, GE Healthcare, Amersham, Buckinghamshire, UK) gradient in RPMI. A 70% Percoll gradient was then layered underneath and centrifuged at 400g for 20 minutes. The fatty layer was removed from the top of the gradient before removing the cell layer below for washing with PBS. Cells were resuspended in Fc block (rat anti-mouse CD16/CD32, 553142, BD Europe, Eysins, Switzerland) at 1:100 for 10 minutes at 4⁰C. Antibodies (Alexa-488-labelled anti-mouse CD11b, 101206, Biolegend, San Diego, California, USA; Pacific Blue-labelled anti-mouse LY-6G, 127612, Biolegend) were added for 30 minutes on ice followed by the addition of lysis fixation solution (349202, BD Europe). Samples were centrifuged at 350g for 5 minutes and resuspended in 200µL PBS prior to analysis. 50µL of Flow-Check^TM^ Fluorospheres (Beckman Coulter, Brea, California, USA) were added to samples and the cytometer (LSR Fortessa, BD Biosciences) was set to collect 10,000 beads. Data were analysed using FlowJo software, version 7.2.4 (Tree Start Inc., Ashland, Oregon, USA).

**Flow Cytometric Lung Digest**

Following exsanguination and retrieval of BALF, lungs were perfused with 10mL PBS through the right ventricle. Lungs were then quickly dissected free and placed in a DNase (DNase 1, 1mg/mL, DN-25, Sigma-Aldrich Company Ltd) and Collagenase (Collagenase D, 10mg/mL, 11088866001, Roche) mix before being disrupted with scissors and incubated at 37⁰C for 1 hour. Cells were further released from tissue by vigorous pipetting using a 1mL syringe and centrifuged at 300g for 15 minutes. The pellet was resuspended in 3mL cold ACK buffer (Ammonium-Chloride-Potassium, A10492-01, Invitrogen, Carlsbad, California, USA) for 5 minutes on ice to lyse red cells before adding 2mL PBS and centrifuged at 300g for 5 minutes. Following another wash, cells were resuspended in PBS and strained using a 40µm cell strainer (352340, BD Biosciences). Fc block^TM^ was added 1:100 to the lung suspension for 10 minutes at 4⁰C prior to another wash in PBS at 300g for 5 minutes. Cells were resuspended in antibody mix in PBS (Alexa-488-labelled anti-mouse CD11b, 101206, Biolegend; Pacific Blue-labelled anti-mouse LY-6G, 127612, Biolegend) and stained on ice for 30 minutes before the addition of lysis fixation solution. Samples were centrifuged at 350g for 5 minutes and resuspended in PBS prior to analysis. The cytometer was set to collect 10,000 events (LSR Fortessa, BD Biosciences). Data analysis was performed using FlowJo software, version 7.2.4 (Tree Star Inc., USA).

**ICAM-1 Expression**

To identify whether neutrophils within the blood and pulmonary interstitium demonstrated signs of reverse transendothelial migration (TEM), ICAM-1 (CD54) expression was assessed 6 hours after receiving FPI. Flow cytometric analysis of blood and lung interstitium were performed using an antibody mix capable of identifying neutrophils and their ICAM-1 expression (Alexa-488-labelled anti-mouse CD11b, 101206, Biolegend; Pacific Blue-labelled anti-mouse LY-6G, 127612, Biolegend; APC-labelled anti-mouse CD54, 561605, BD Biosciences, UK).

**Proteome Profiler Array**

Proteome profiler kit for the measurement of 111 different cytokines in BAL fluids (Proteome Profiler Mouse XL Cytokine Array, R&D Systems) was used according to the manufacturers’ instructions.

**Flow Cytometric Analysis of Blood**

To assess leukocyte mobilisation in the blood, 20μL of whole blood was obtained via the tail vein and immediately mixed with 3.9% sodium citrate (1:1). Blood was aliquoted into flow cytometry tubes and antibodies (mouse) were added (Pacific Blue-labelled anti-mouse LY-6G, 127612, Biolegend; PE- labelled anti-mouse CD45, 103106, Biolegend) for 30 minutes on ice. Red blood cells (RBCs) were then lysed using FACs lysing solution (349202, BD Europe). Samples were centrifuged at 350g for 5 minutes and resuspended in PBS prior to analysis. 50μL of Flow-CheckTM Fluorospheres (Beckman Coulter, High Wycombe, UK) were added to samples and the cytometer was set to collect 10,000 beads. Analysis was performed on a BD LSR Fortessa flow cytometer. Data were analysed using FlowJo software, version 7.2.4 (Tree Star Inc., USA).

**SUPPLEMENTARY LEGENDS**

Supplementary Figure E1: **Outline of Experimental Protocols.** A) Fluid Percussion Injury experimental protocol. B) Timeline of Fluid Percussion Injury experiments. Mice received 1.5ATM FPI or SHAM injury, and were retrieved at 6, 24 and 48 hours. C) Timeline of HCl Double Hit. 50μL pH 1.75 HCl was instilled via an *intra-tracheal* route immediately after FPI/SHAM procedure and lungs retrieved 6 hours later. D) Timeline of HCl Double Hit with anti-LY-6G. Mice were pre-injected with anti-LY-6G 24 hours prior to receiving FPI-HCl. BALF was retrieved 6 hours after FPI.

Supplementary Figure E2: **Righting Time following FPI/SHAM treatment**. An exclusion time of 270 seconds following FPI was used. Mice receiving FPI (n=45) took significantly longer to right than mice receiving SHAM (n=47). Data are represented as mean ± SD and were analysed using the student’s t-test (***p<0.001).

Supplementary Figure E3: **Histological and Flow Cytometric Analysis of Fluid Percussion Induced Brain Injury.** Individual hemispheres were digested 6, 24 and 48 hours after receiving FPI/SHAM procedures for flow cytometry or processed for histology. A) Representative brain sections stained with acid fuchsin 24 hours after SHAM/FPI treatment. Homogenous staining can be seen following SHAM treatment however arrow points to intensely stained irreversibly damaged neurons following FPI. B) Myeloperoxidase immunohistochemistry on brain sections 24 hours after SHAM/FPI procedure. Arrows point to DAB+ve cells (neutrophils) located in areas of neuronal damage in proximity to the craniotomy/injury site. C) Representative flow plots of the ipsilateral hemisphere 6 hours after SHAM/FPI procedure. Neutrophils were identified as CD11b+ve, LY-6G+ve. Scale bar represents 100μm.

Supplementary Figure E4: **Cytocentrifuge preparations following Concussion TBI.** Cytocentrifuge preparations were generated from BALF collected 6, 24 and 48 hours after FPI/SHAM procedures showing only the presence of alveolar macrophages with no alveolar neutrophils. Scale bar represents 100μm.

Supplementary Figure E5**: BALF Analysis following Concussive FPI.** BALF was collected 6, 24 and 48 hours after FPI/SHAM procedure to assess signs of pulmonary injury/inflammation. A) BALF Cell Count. No differences were seen. B) BALF IL-1β Expression. Although noticing a drop in IL-1β at 24 hours, no significant differences were observed in alveolar IL-1β expression. C) BALF Total Protein. No differences in BALF total protein were observed after FPI. Data are represented as mean ± SD and were analysed using 2-way ANOVA (n=3-6/group, *p<0.05).

Supplementary Figure E6**: Interstitial Neutrophil Infiltration following FPI.** Interstitial lung tissue was collected 6, 24 and 48 hours after FPI/SHAM procedure to assess signs of pulmonary injury/inflammation. A) Representative flow plots of pulmonary interstitium 6 hours after SHAM/FPI treatment. Neutrophils (CD11b+ve, LY-6G+ve) were seen to increase following FPI. B) Myeloperoxidase (MPO) immunohistochemistry on lung sections 6 hours after SHAM/FPI procedure. Significantly more neutrophils (DAB+ve) were seen 6 hours after FPI, which confirmed the findings seen with flow cytometry. Scale bar represents 100μm.

Supplementary Figure E7**: Neutrophil ICAM-1 Expression within the Bloodstream and Pulmonary Interstitium.** A) Neutrophil ICAM-1 Expression. A non-significant drop in ICAM-1 expression was observed after FPI/SHAM procedure. B) Interstitial Neutrophil ICAM-1 Expression. No differences were seen in neutrophil ICAM-1 expression following FPI/SHAM procedure. Data represented as mean +/- SD. Data analysed using the students t-test (n=3/group).

Supplementary Figure E8**:** **Proteome Profiler Array of SHAM and FPI BALF (6 hour).** BALF was retrieved 6 hours after SHAM/FPI procedure and the expression of 111 cytokines was assessed. FPI induced increased expression of most cytokines assessed. Line represents no relative change, with any samples located above the line showing increased expression following FPI. Data represented as mean pixel intensity.

Supplementary Figure E9**: Neutrophil depletion with anti-LY-6G.** Representative flow plots of pulmonary interstitium 24 hours after isotype control/anti-LY-6G depleting antibody. Neutrophils (CD11b+ve, LY-6G+ve) could still be identified following isotype control however anti-LY-6G successfully depleted neutrophils within the lung.

Supplementary Figure E10: **BALF Analysis following HCl Double Hit with Anti-LY-6G.** Mice were pre-injected with anti-LY-6G 24 hours prior to receiving FPI-HCl. BALF was retrieved 6 hours after FPI. A) BALF Macrophages. Anti-LY-6G had no effect on macrophage number. B) BALF Cytokine Array. Tumor Necrosis Factor (TNF), Monocyte Chemoattractant Protein-1 (MCP-1) and IL-6 levels were shown to be elevated in FPI-HCl treated mice compared to SHAM-HCl. Neutrophil depletion resulted in a mild reduction in TNF and IL-6 levels however had no effect on MCP-1 levels. Data are represented as mean ± SD and were analysed using 1-way ANOVA and student’s t-test (n=3-6/group).

Supplementary Table E1**: Summary of Proteome Profiler Array of SHAM and FPI BALF (6 hour).** BALF was retrieved 6 hours after SHAM/FPI procedure and the expression of 111 cytokines was assessed. Pooled samples from n=3 per group were analysed on the proteome profiler. FPI induced increased expression of most cytokines assessed. Many cytokines capable of inducing neutrophil chemotaxis (CCL2/MCP-1, CXCL1/KC, CXCL2/MIP-2, IL-6, TNF) were upregulated following FPI.

Supplementary Table E1**: Summary of Proteome Profiler Array of SHAM and FPI BALF (6 hour).**

| **Protein** | **SHAM** | **FPI** |
| --- | --- | --- |
| Adiponectin/Acrp30 | 25.7925 | 88.428 |
| Amphiregulin | 0 | 6.482 |
| Angiopoietin-1 | 0 | 5.9545 |
| Angiopoietin-2 | 0 | 10.567 |
| Angiopoietin-like 3 | 3.838 | 27.5 |
| BAFF/BLyS/TNFSF13B | 21.7065 | 35.3285 |
| C1q R1/CD93 | 0 | 25.6435 |
| CCL2/JE/MCP-1 | 0 | 5.2795 |
| CCL3/CCL4 MIP-1 alpha/beta | 0 | 4.0585 |
| CCL5/RANTES | 0 | 4.6525 |
| CCL6/C10 | 100.0265 | 113.4005 |
| CCL11/Eotaxin | 1.4775 | 6.937 |
| CCL12/MCP-5 | 8.207 | 21.3825 |
| CCL17/TARC | 0 | 20.1575 |
| CCL19/MIP-3 beta | 0 | 7.626 |
| CCL20/MIP-3 alpha | 4.851 | 9.5625 |
| CCL21/6Ckine | 0.671 | 40.0675 |
| CCL22/MDC | 0 | 9.1345 |
| CD14 | 16.973 | 10.716 |
| CD40/TNFRSF5 | 0 | 7.558 |
| CD160 | 3.536 | 17.0675 |
| Chemerin | 3.806 | 28.45 |
| Chitinase 3-like 1 | 52.3195 | 65.063 |
| Coagulation Factor III/Tissue Factor | 25.77 | 32.2565 |
| Complement Component C5/C5a | 22.937 | 9.018 |
| Complement Factor D | 52.6435 | 60.896 |
| C-Reactive Protein/CRP | 8.986 | 26.3965 |
| CX3CL1/Fractalkine | 8.5625 | 8.261 |
| CXCL1/KC | 0.612 | 10.1215 |
| CXCL2/MIP-2 | 0 | 4.0805 |
| CXCL9/MIG | 0 | 11.162 |
| CXCL10/IP-10 | 0.5765 | 8.6935 |
| CXCL11/I-TAC | 0 | 7.0085 |
| CXCL13/BLC/BCA-1 | 0 | 9.455 |
| CXCL16 | 27.977 | 30.117 |
| Cystatin C | 26.279 | 30.7655 |
| Dkk-1 | 0 | 6.2655 |
| DPPIV/CD26 | 28.725 | 33.441 |
| EGF | 14.279 | 46.9415 |
| Endoglin/CD105 | 0 | 6.842 |
| Endostatin | 10.527 | 49.288 |
| Fetuin A/AHSG | 36.4455 | 43.1665 |
| FGF acidic | 63.1485 | 85.7835 |
| FGF-21 | 1.725 | 11.6665 |
| Flt-3 Ligand | 11.2925 | 26.0585 |
| Gas6 | 25.225 | 26.351 |
| G-CSF | 0 | 8.1485 |
| GDF-15 | 2.004 | 10.2925 |
| GM-CSF | 0 | 6.9995 |
| HGF | 4.1525 | 14.6395 |
| ICAM-1/CD54 | 51.905 | 54.378 |
| IFN-gamma | 0 | 6.2835 |
| IGFBP-1 | 0 | 11.536 |
| IGFBP-2 | 41.923 | 53.3645 |
| IGFBP-3 | 10.986 | 58.824 |
| IGFBP-5 | 3.1755 | 22.0225 |
| IGFBP-6 | 41.9545 | 56.1395 |
| IL-1 alpha/IL1F1 | 3.351 | 9.153 |
| IL-1 beta/IL-1F2 | 0.3195 | 7.2925 |
| IL-1ra/IL-1F3 | 2.324 | 7.7385 |
| IL-2 | 0 | 5.351 |
| IL-3 | 0 | 7.5535 |
| IL-4 | 1.5855 | 11.5585 |
| IL-5 | 2.216 | 9.076 |
| IL-6 | 0 | 4.2745 |
| IL-7 | 4.8375 | 9.82 |
| IL-10 | 0.9185 | 12.342 |
| IL-11 | 0.1395 | 10.1395 |
| IL-12p40 | 0.2025 | 10.3425 |
| IL-13 | 0 | 6.2205 |
| IL-15 | 0.63 | 7.189 |
| IL-17A | 0 | 4.2385 |
| IL-22 | 0.36 | 6.6395 |
| IL-23 | 0.2425 | 5.387 |
| IL-27 | 4.0945 | 10.878 |
| IL-28 | 19.1305 | 22.599 |
| IL-33 | 0.833 | 7.3375 |
| LDL R | 15.6305 | 17.189 |
| Leptin | 0 | 6.797 |
| LIF | 0.1755 | 8.554 |
| Lipocalin-2/NGAL, | 18.9775 | 24.5135 |
| LIX | 11.9995 | 8.5495 |
| M-CSF | 1.4455 | 10.3825 |
| MMP-2 | 4.8465 | 31.0225 |
| MMP-3 | 0.0765 | 8.5985 |
| MMP-9 | 0 | 7.1035 |
| Myeloperoxidase | 3.734 | 16.3065 |
| Osteopontin (OPN) | 56.8335 | 33.2115 |
| Osteoprotegerin/TNFRSF11B | 0 | 5.3465 |
| PD-ECGF/Thymidine phosphorylase | 0 | 4.18 |
| PDGF-BB | 4.414 | 13.7115 |
| Pentraxin 2/SAP | 13.869 | 22.5945 |
| Pentraxin 3/ TSG-14 | 5.112 | 9.4865 |
| Periostin/OSF-2 | 1.572 | 14.157 |
| Pref-1/DLK-1/FA1 | 5.3915 | 12.243 |
| Proliferin | 5.144 | 10.1845 |
| Proprotein Convertase 9/PCSK9 | 2.3465 | 20.423 |
| RAGE | 46.045 | 57.833 |
| RBP4 | 29.689 | 34.621 |
| Reg3G | 80.144 | 74.0985 |
| Resistin | 4.3735 | 15.725 |
| E-Selectin/CD62E | 0.4595 | 11.297 |
| P-Selectin/CD62P | 0.3195 | 14.1435 |
| Serpin E1/PAI-1 | 37.6935 | 59.725 |
| Serpin F1/PEDF | 57.9505 | 71.9325 |
| Thrombopoietin | 0 | 7.279 |
| TIM-1/KIM-1/HAVCR | 0 | 6.441 |
| TNF | 0 | 4.7745 |
| VCAM-1/CD106 | 5.0535 | 34.2925 |
| VEGF | 11.9995 | 19.428 |
| WISP-1/CCN4 | 0 | 8.617 |

**Supplementary Material References**

1. Carbonell WS, Maris DO, McCall T, Grady MS. Adaptation of the fluid percussion injury model to the mouse. *J Neurotrauma* 1998;15:217–229.
