# Supplementary figures and images for: Cerebral Concussion Primes the Lungs for Subsequent Neutrophil-Mediated Injury
Source: Crit Care Med. 2018 Aug 15;46(9):e937–44. doi: 10.1097/CCM.0000000000003270 (PMC6110623; doi:10.1097/CCM.0000000000003270)

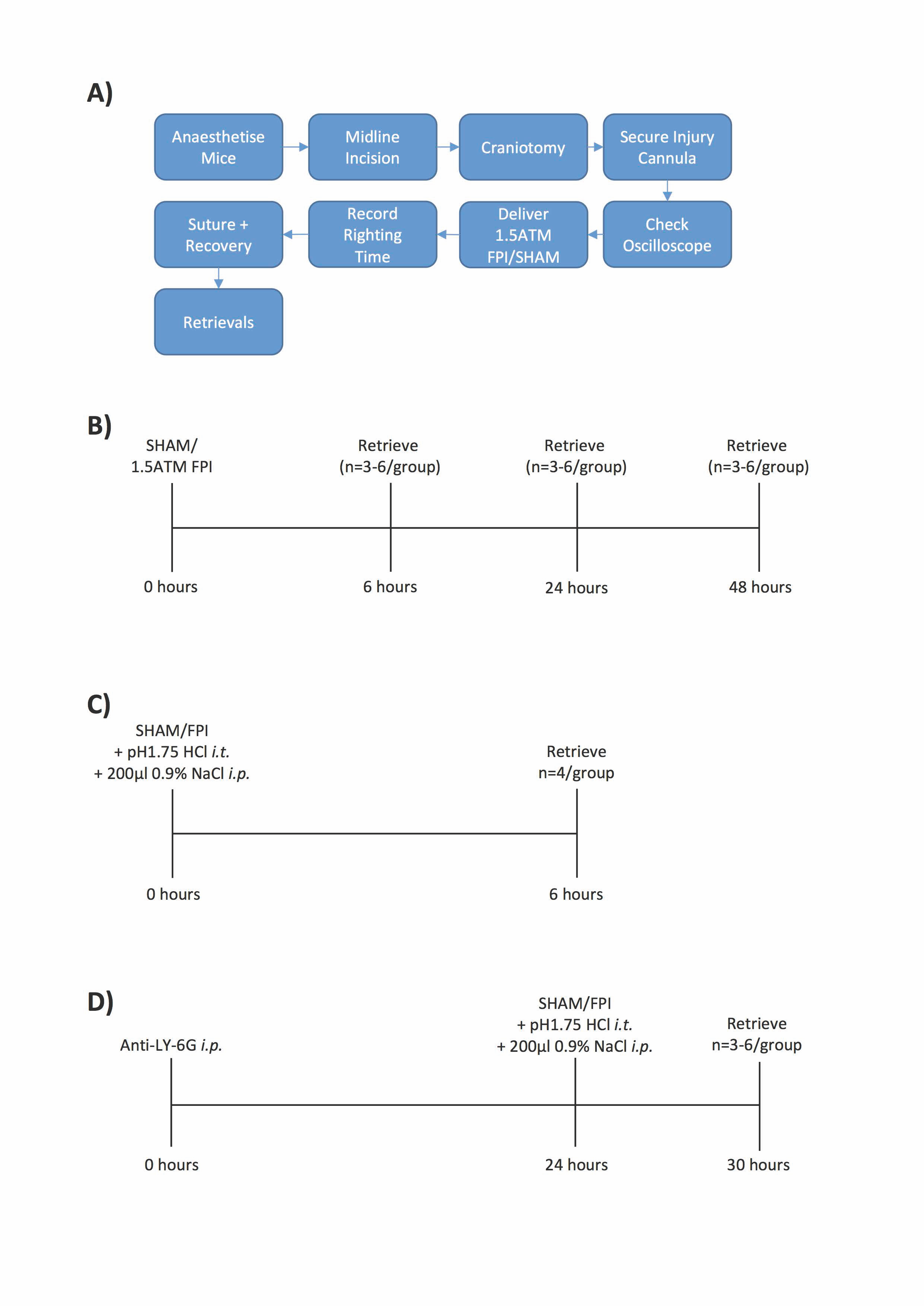

Supplement: Supplementary file 2 [file ccm-46-e937-s002.tif]

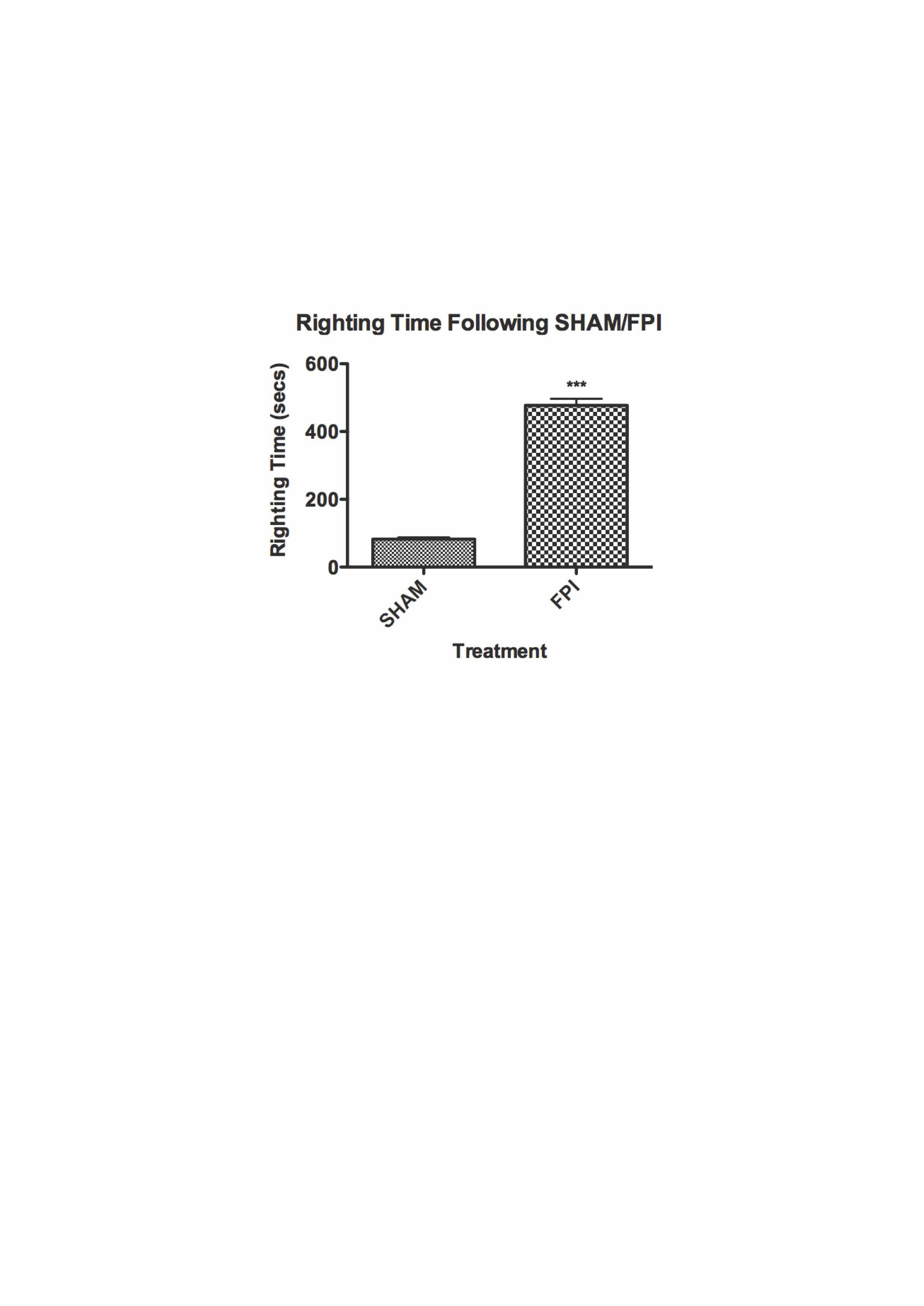

Supplement: Supplementary file 4 [file ccm-46-e937-s004.tif]

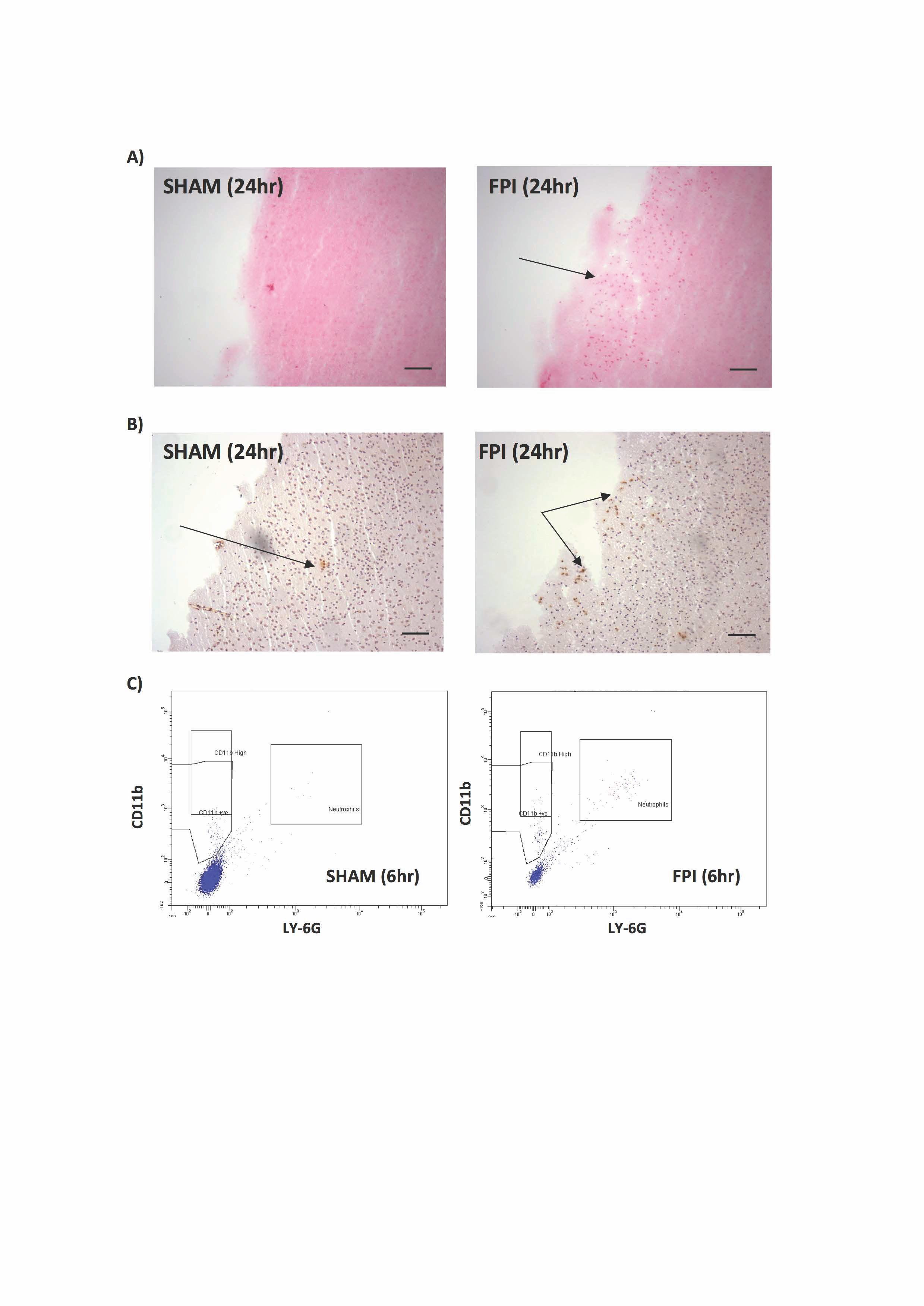

Supplement: Supplementary file 5 [file ccm-46-e937-s005.tif]

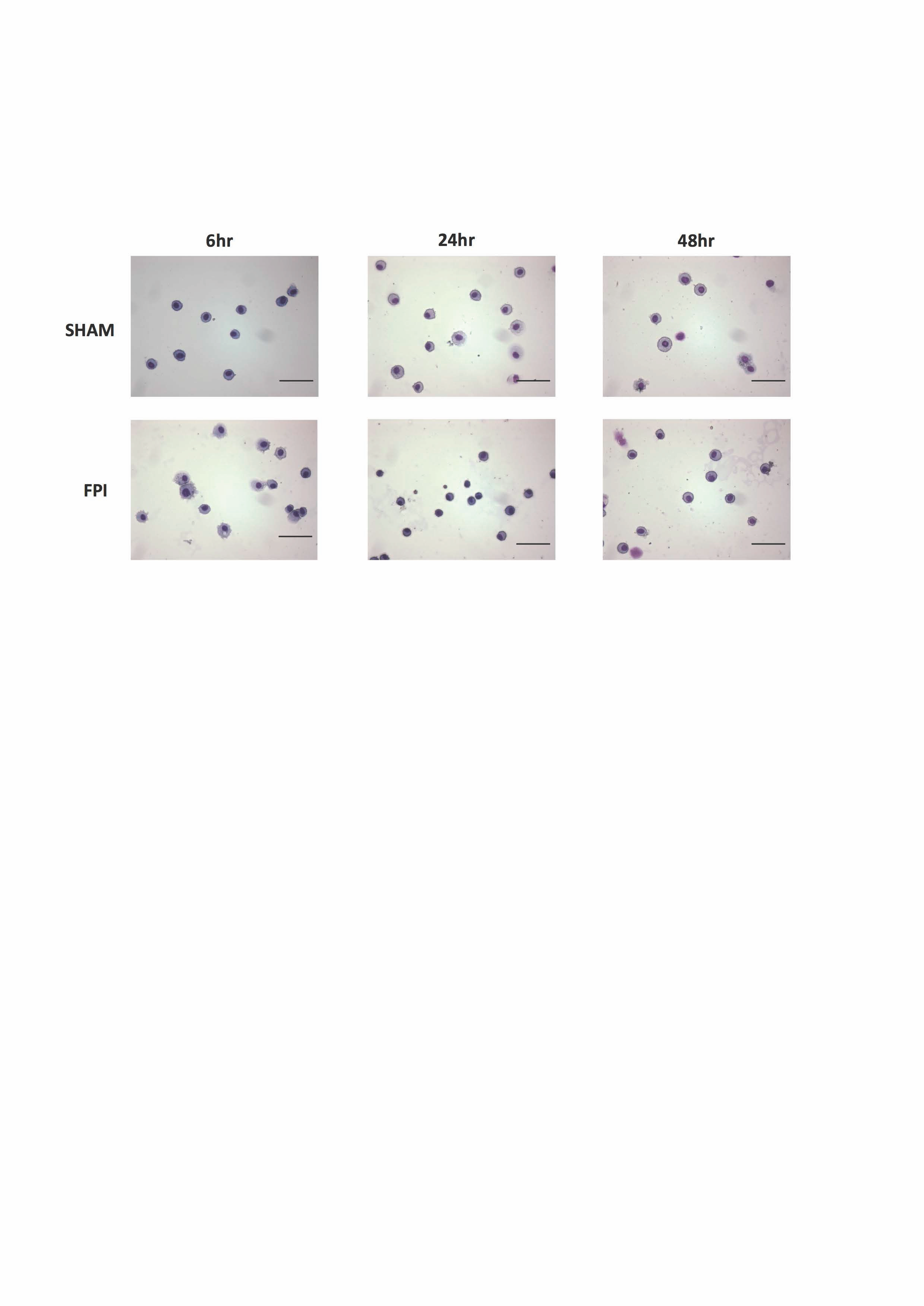

Supplement: Supplementary file 6 [file ccm-46-e937-s006.tif]

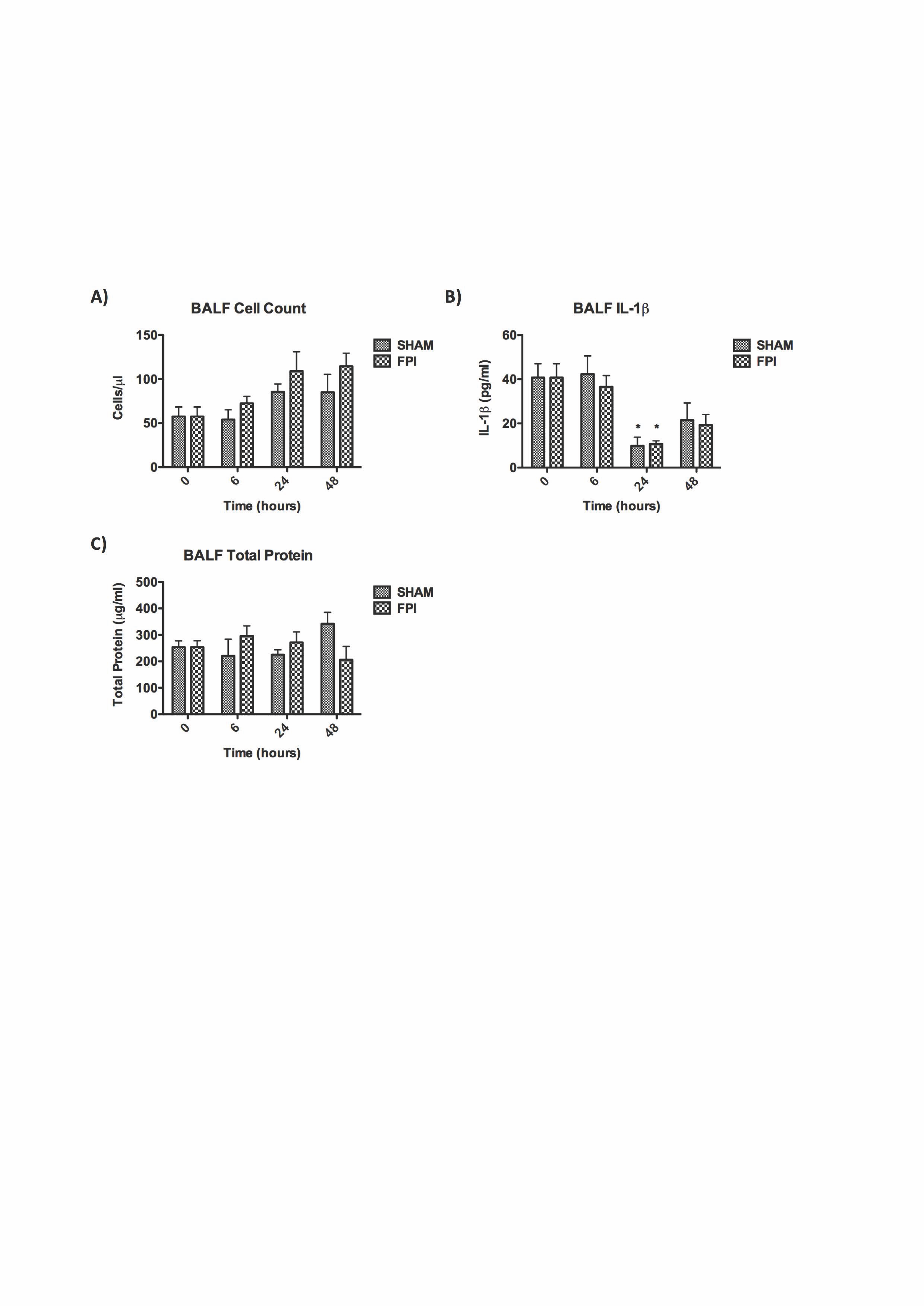

Supplement: Supplementary file 7 [file ccm-46-e937-s007.tif]

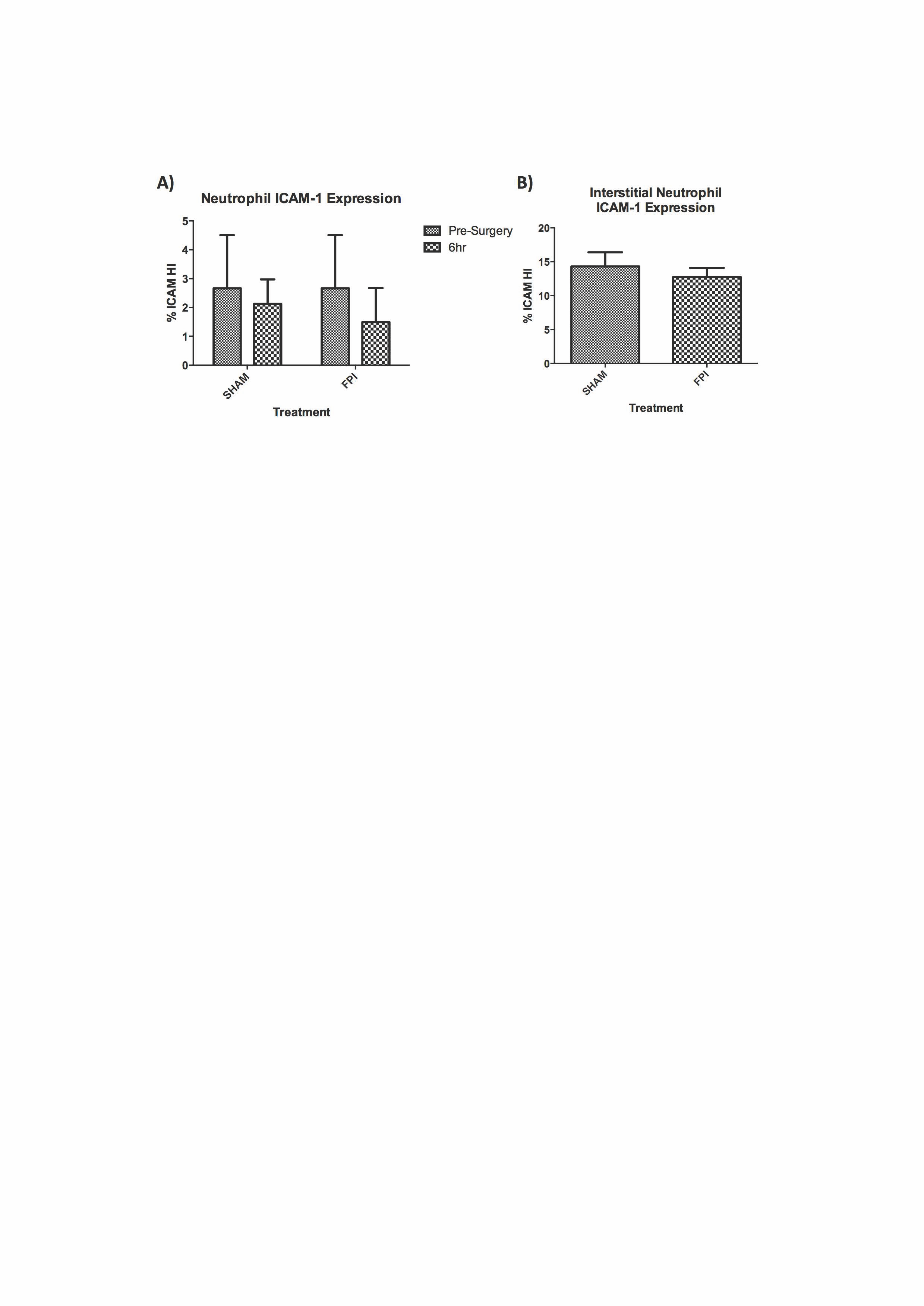

Supplement: Supplementary file 8 [file ccm-46-e937-s008.tif]

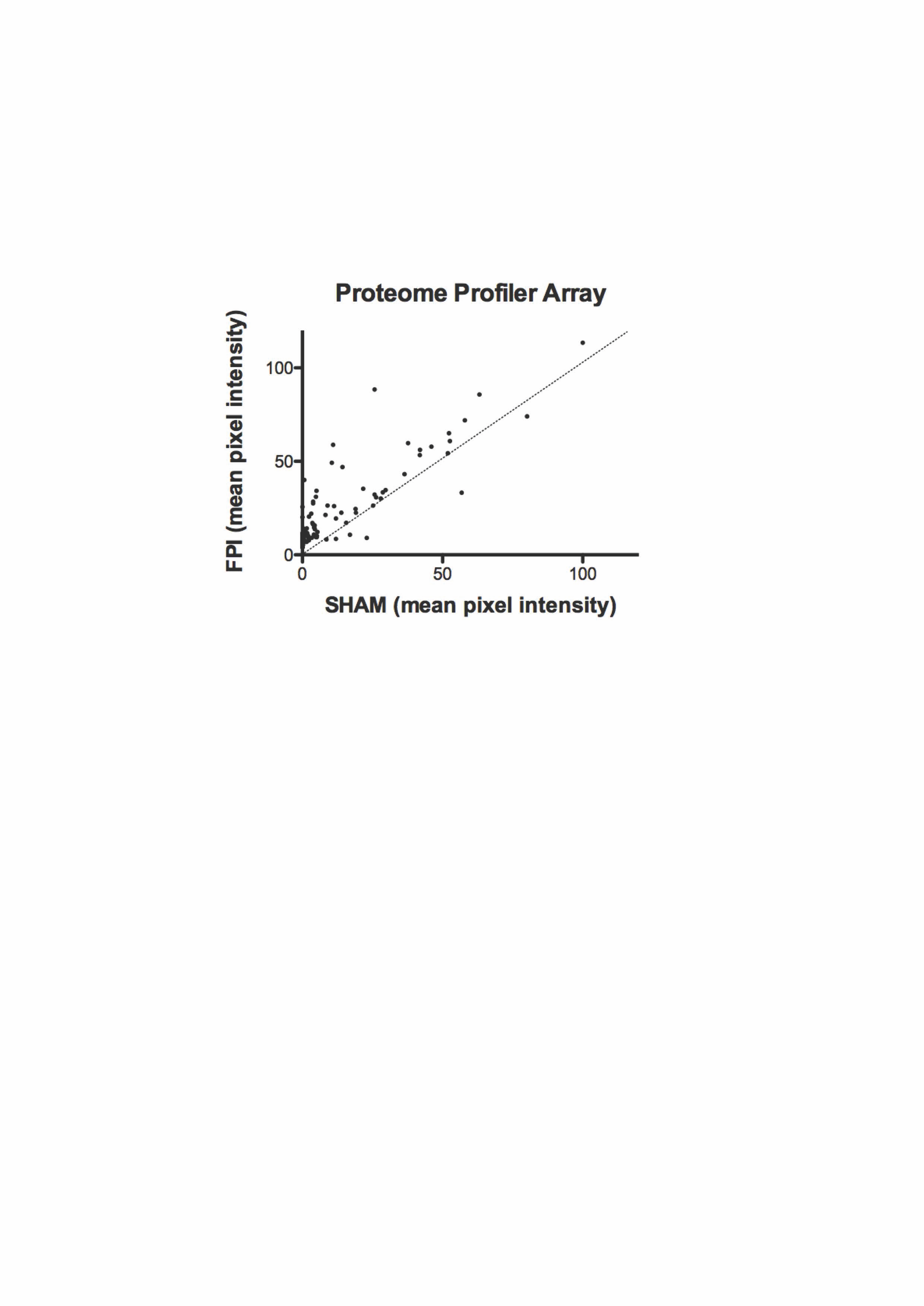

Supplement: Supplementary file 9 [file ccm-46-e937-s009.tif]

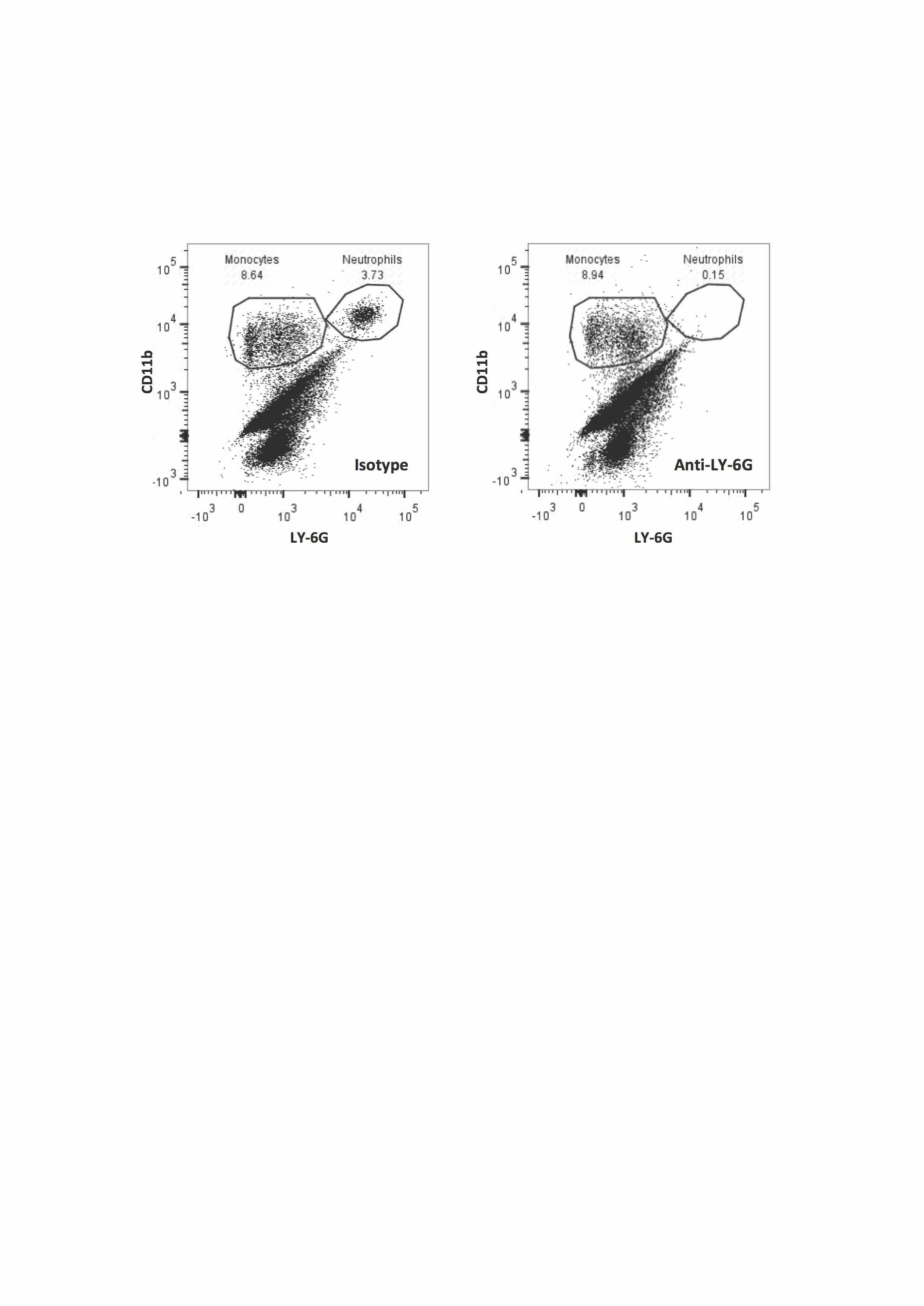

Supplement: Supplementary file 10 [file ccm-46-e937-s010.tif]

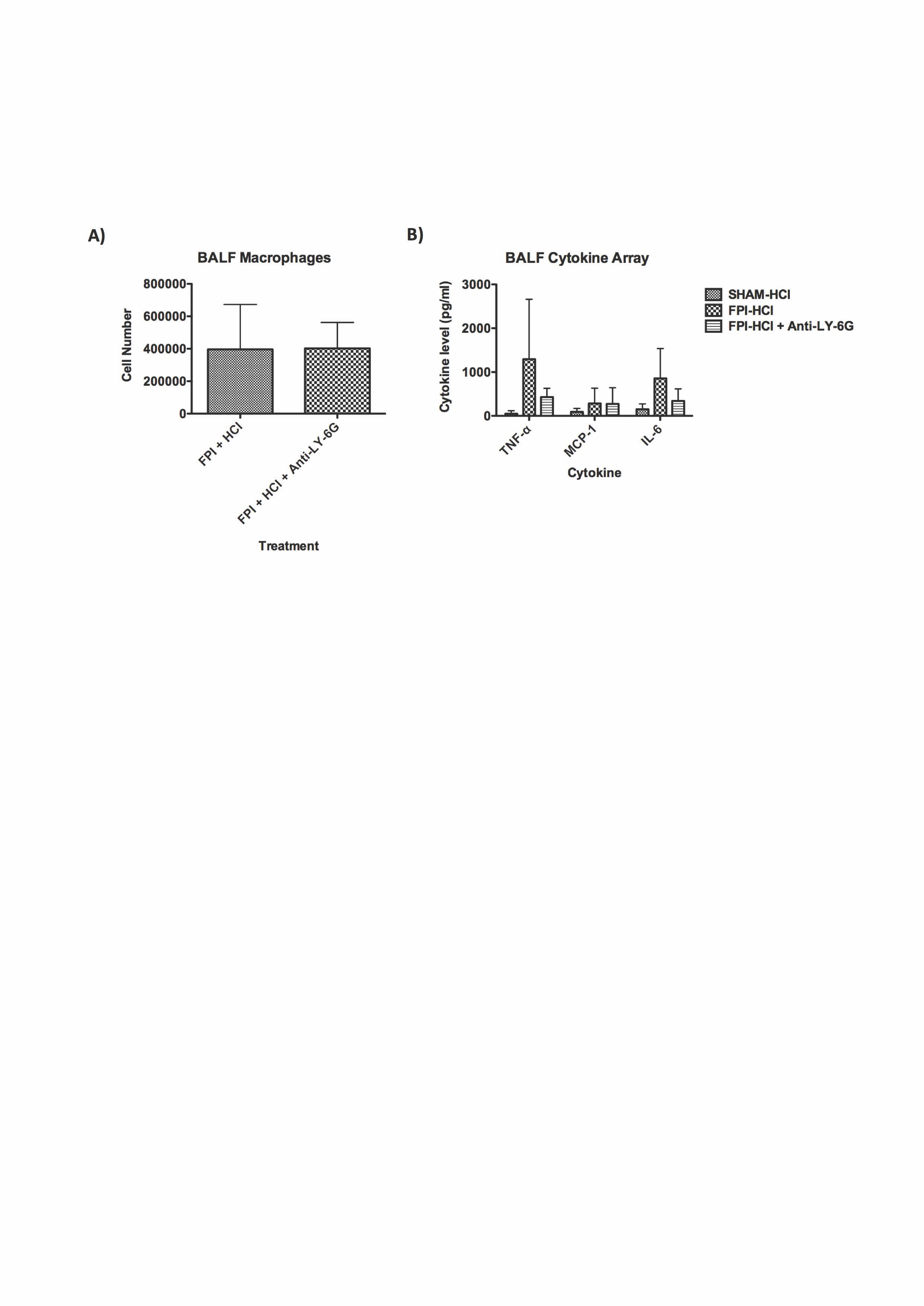

Supplement: Supplementary file 11 [file ccm-46-e937-s011.tif]
