# Supplementary material for: Cerebral Concussion Primes the Lungs for Subsequent Neutrophil-Mediated Injury
Source: Crit Care Med. 2018 Aug 15;46(9):e937–44. doi: 10.1097/CCM.0000000000003270 (PMC6110623; doi:10.1097/CCM.0000000000003270)
Supplement: Supplementary file 3 [file ccm-46-e937-s003.docx]

**Supplemental Methods**

**Description of data collection**

Relevant clinical data were collected at the time of hospitalization (refer to supplementary methods for description of variables). , including: gestational age, birth weight, gender, ethnicity (Dutch/≥1 non-native Dutch parent), inborn, the need for ECMO, Pediatric Logistic Organ Dysfunction-2(PELOD-2) score(16) in the first 24 hours of pediatric intensive care unit(PICU) stay (or up to ECMO cannulation in ECMO-treated patients if ECMO was initiated in the first 24 hours of PICU stay), the maximum vasoactive-inotropic score (VIS)(17) was recorded up until ECMO cannulation for the ECMO-treated patients or up until hernia repair for the CDH-non-ECMO patients, calculated as follows: dopamine dose (ug/kg/min)+dobutamine dose (ug/kg/min)+(100xepinephrine dose (ug/kg/min))+10xmilrinone dose (ug/kg/min)+(10,000xvasopressin dose (U/kg/min))+(100xnorepinephrine dose (ug/kg/min)), cardiopulmonary resuscitation(CPR) during initial hospital stay, sepsis during initial hospital stay (clinical suspicion of sepsis with positive blood culture), duration of initial mechanical ventilation, ventilator-free days in the first 28 days of life, duration of PICU stay, duration of initial hospitalization, pulmonary hypertension on echocardiography during PICU admission, inhaled nitric oxide requirement, sildenafil requirement, the presence of chronic lung disease(oxygen dependency at 28 days of life)(18), and number of anesthetic procedures in the first year of life (including CDH repair and/or ECMO (de)cannulation). Additional characteristics for ECMO patients included: highest oxygenation index before ECMO, age at start ECMO, ECMO type, ECMO duration, and cranial ultrasound result before and after ECMO. Additional data for CDH patients were: diaphragmatic defect side, surgical repair technique (thoracoscopy or laparotomy), age at surgery, and patch repair requirement.
